# High-density genetic map construction and QTL mapping to identify genes for blight defense- and yield-related traits in sesame (*Sesamum indicum* L.)

**DOI:** 10.3389/fpls.2024.1446062

**Published:** 2024-09-26

**Authors:** Guizhen Xu, Yanqin Cui, Sida Li, Zhongbo Guan, Hongmei Miao, Yuanzhang Guo

**Affiliations:** ^1^ Hebei Laboratory of Crop Genetics and Breeding, Institute of Cereal and Oil Crops, Hebei Academy of Agriculture and Forestry Sciences, Shijiazhuang, China; ^2^ Henan Sesame Research Center, Henan Academy of Agricultural Sciences, Zhengzhou, China

**Keywords:** *Sesamum indicum* L, genetic map, QTL mapping, fusarium wilt disease, yield-related traits

## Abstract

Sesame (*Sesamum indicum* L.) is an important oilseed crop widely cultivated in subtropical and tropical areas. Low genetic yield potential and susceptibility to disease contribute to low productivity in sesame. However, the genetic basis of sesame yield- and disease-related traits remains unclear. Here, we represent the construction of a high-density bin map of sesame using whole genome sequencing of an F2 population derived from ‘Yizhi’ and ‘Mingdeng Zhima’. A total of 2766 Bins were categorized into 13 linkage groups. Thirteen significant QTLs were identified, including ten QTLs related to yield, two QTLs related to Sesame Fusarium wilt (SFW) disease, and one QTL related to seed color. Among these QTLs, we found that SFW-QTL1.1 and SFW-QTL1.2 were major QTLs related to Fusarium wilt disease, explaining more than 20% of the phenotypic variation with LOD > 6. SCC-QTL1.1 was related to seed coat color, explaining 52% of the phenotypic variation with LOD equal to 25.3. This suggests that seed color traits were controlled by a major QTL. Candidate genes related to Fusarium wilt disease and seed color in the QTLs were annotated. We discovered a significant enrichment of genes associated with resistance to late blight. These genes could be spectral disease resistance genes and may have a role in the regulation of Fusarium wilt disease resistance. Our study will benefit the implementation of marker-assisted selection (MAS) for the genetic improvement of disease resistance and yield-related traits in sesame.

## Introduction

Sesame (*Sesamum indicum* L.), an indispensable oilseed crop renowned for its versatility and nutritional richness, belongs to the genus *Sesamum*, nestled within the expansive Pedaliaceae family of flowering plants. Sesame seeds have a high oil content, ranging from 50% to 60%. They also contain multiple biologically active values of sesame peptides, making them a healthy and nutritious oil crop (Wei et al., 2022). The oil extracted from sesame seeds, known as sesame oil, has a pleasant fragrance. It serves multiple purposes, including being used as a direct food ingredient, a base for ointments, a lubricant, and a detoxifier. Additionally, sesame is abundant in natural antioxidants such as sesamin, sesamol, and sesaminol. These antioxidants have the potential to be utilized as active ingredients in a range of products, such as preservatives, disinfectants, antiviral agents, insecticides, and anti-tuberculosis agents (Mili et al., 2021; Wei et al., 2022). Despite its excellent characteristics, sesame remains under-researched compared to other crops. This is one of the reasons for its low and unstable yield. Since sesame seeds are the primary raw material for production, enhancing the yield of sesame seeds is a subject of concern in both agricultural production and scientific research.

The growth cycle of sesame requires high temperature and high humidity, making it highly susceptible to various pathogenic fungi and resulting in multiple sesame diseases (Chinchilla et al., 2009). Stem rot disease and wilt disease are considered the most serious diseases in sesame production (Kwon et al., 2013; Zhang et al., 2020). *Sclerotinia sclerotiorum* and *Fusarium oxysporum* are the pathogens responsible for stem rot disease and wilt disease, respectively (Duan et al., 2020; Aguilar-Pérez et al., 2022). Current approaches to plant disease defense involve the use of chemical pesticides and improved cultivation methods. However, these methods are costly and fail to address the root causes of plant diseases. Biotechnological approaches, utilizing molecular biology techniques, have emerged as a precise and expedited methods for enhancing disease resistance in plants. Plants primarily employ pathogen-associated molecular patterns (PAMPs)-triggered immunity and effector-triggered immunity in response to pathogen invasion (Asai et al., 2002; Boller and He, 2009; Chinchilla et al., 2009). The CaAMP1 gene enhances the resistance to stem wilt disease in soybeans, while ZmWRKY83 enhances resistance to *Fusarium graminearum* rot infection in maize (Niu et al., 2020; Bai et al., 2021). Although plant-pathogen interaction pathways have been established in model plants, there is still a significant amount of unknown information.

Currently, significant progress has been made in sequencing and assembly of high-quality plant genomes, providing necessary gene sequence references for genome resequencing (Huang et al., 2009; Li et al., 2020; Sun et al., 2022). Genome resequencing provides a large number of variant sites, such as single nucleotide polymorphisms (SNPs), copy number variations (CNVs), and insertion/deletion (InDel) mutations, enabling efficient and accurate acquisition of genetic characteristics of biological populations (Huang et al., 2009; Zegeye et al., 2018). Analyzing the genetic characteristics of biological populations reveals the population’s evolutionary history, gene flow, and adaptation to environmental changes. This understanding helps us comprehend the mechanisms and patterns of biological evolution (Chen and Narum, 2021; North et al., 2021; Wang et al., 2022). These genetic characteristics can assess the species adaptability, genetic health status, and degree of endangerment, leading to the development of effective conservation strategies and promoting biodiversity protection and sustainable utilization (Chen and Narum, 2021; Wang et al., 2022). Moreover, they can identify genes with beneficial traits, improving crop varieties, enhancing yield, resistance, and other economic traits, and promoting sustainable agricultural development (Huang et al., 2009; Zegeye et al., 2018; Pawlowski et al., 2020). Obtaining the genetic characteristics of biological populations provides fundamental data and references for scientific research and practical applications in plant science, with wide-ranging applications in plant research.

## Materials and methods

### Plant materials and growing environment

In total, 169 individuals and two parents (‘Yizhi’ and ‘Mingdeng Zhima’) were collected for genome resequencing. Plants were grown on sandy or loamy soil with medium to high fertility. Plants were 16.5 cm apart with a row-to-row distance of 40 cm, totaling 10,000 plants per acre.

### Collection and analysis of phenotypic data

Data were collected on eleven phenotypic traits, including plant height (PH), first capsule height (CH), capsules length (CL), infertile top length (ITL), seeds per capsule (SPC), capsules per plant (CPP), 1000-seed weight (SW), yield per plant (YPP), number of capsule layers (NCL), sesame fusarium wilt (SFW) and seed coat color (SCC). To determine the phenotypic traits of sesame more accurately, we divided its growth cycles into ten stages, as shown in **Supplementary Table S11**. The criteria for the measurement of 11 phenotypic traits are shown in **Supplementary Table S12**. Three biological replicates were recorded.

### Genomic DNA extraction and genotyping

Young leaves were collected from parental plants and F2 progeny in liquid nitrogen and stored at −80°C. The plant DNA extraction Kit (Tiangen Biotech, Beijing, China) was used to extract the genomic DNA. The quality of the isolated DNA was checked using agarose gel electrophoresis and assessed using a Qubit 2.0 Fluorometer (Thermo Fisher, CA, USA). Paired-end sequencing libraries with 150 bp insert-sizes were constructed. Over 10 Gb clean data were generated for each sample using the Illumina Casava 1.8 platform. The parental DNA samples were replicated to detect SNPs and estimate marker segregation types. The raw sequencing data were filtered to get high-quality clean reads. Clean reads of all samples of parents and offspring were mapped to the reference genome of *S. indicum* Zhongzhi13_v2.0 using Burrows-Wheeler Aligner (BWA) v0.7.10. Calibrated alignments were used to call genomic variants using the HaplotypeCaller Genome Analysis Toolkit (GATK) v4.2. The raw SNPs and Indel variants were filtered using the following parameters: ‘QD < 2.0 || MQ < 40.0 || FS > 60.0 || QUAL <30.0 ||-clusterSize 2-clusterWindowSize 5’. The identified SNPs and indels were annotated using SnpEff v4.3T tool software.

### Linkage map construction using the F2 population

To ensure the accuracy of the linkage map, we selected markers that were homozygous and inconsistent between parents with a sequencing depth of more than four and removed non-chromosomal markers. Markers with more than 30% missing data were filtered. A total of 110345 SNPs were obtained to construct linkage map. Chromosome number and order of markers were anchored according to the physical map. HighMap was used to estimate the genetic distance between markers (Liu et al., 2014). The process involved: (1) data input and preprocessing; (2) Identifying linkage groups; (3) sorting and reordering markers to optimize their positions along the linkage groups; (4) performing genotype error correction to further improve the genetic map’s quality; (5) estimating the genetic distance between markers based on the corrected marker order; (6) Assessing the quality of the genetic map. Recombination frequency was converted into map distance by the Kosambi mapping function. R package LinkageMapView was used for visualization of the linkage map.

### QTL mapping for fusarium wilt defense- and yield-related traits

The algorithm of composite interval mapping (CIM) for the F2 population implemented by R/qtl was used for QTL mapping of the eleven traits. The LOD threshold was set at 3.0. The scanning step was 0.5 cM. The two probabilities for entering and removing variables were set at 0.001 and 0.002, respectively. Comparison of QTL mapping results among the eleven traits was conducted. QTLs in different traits were considered to be common if the genetic positions were close enough (linkage map was less than 20 cM in terms of QTL positions. ShinyCircos was used for the visualization of QTL positions on the linkage map.

### Candidate gene identification and annotation

We detected the genes in the QTL region based on the functional annotation of *S. indicum* reference genome Zhongzhi13_v2.0 (Basak et al., 2019). The gene annotations were screened for polymorphism at the amino acid level between the re-sequenced parents. Genes showing more than one changes at the amino acid level were considered candidates. For detailed functional annotation, we compared the candidates with the NR protein sequences available at UniProt database using the BLASTX algorithm, with an E-value threshold of 10^-01^. The associated hits were then searched for their respective Gene Ontology (GO) terms at www.geneontology.org and Kyoto Encyclopedia of Genes and Genomes (KEGG) pathway at https://www.genome.jp/kegg/.

## Results

### Phenotypic evaluation and correlation analysis among eleven traits

**Figure 1:**
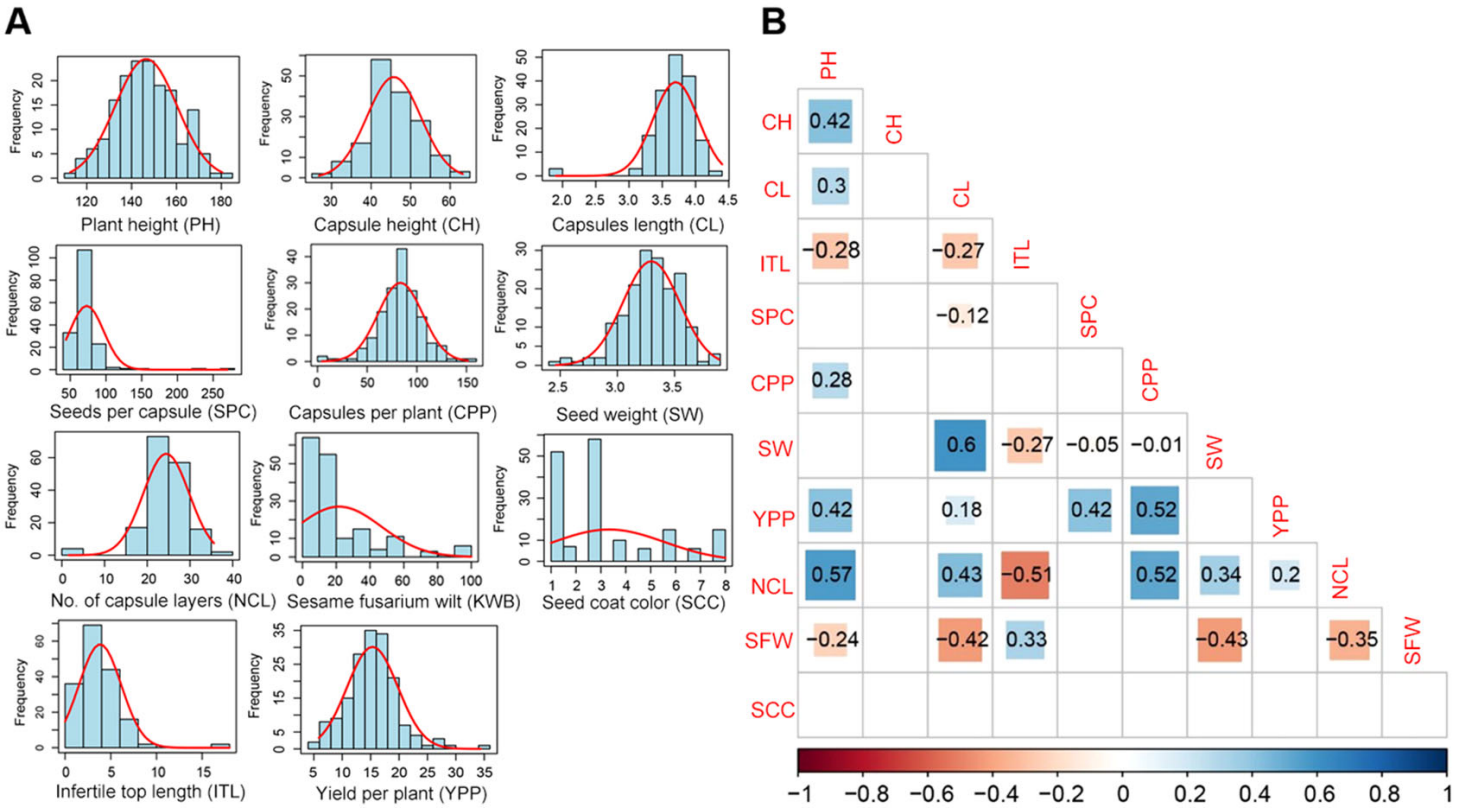
Analysis of eleven traits related to growth, yield, and disease resistance. **(A)** Histograms for the frequency distribution of the traits. **(B)** Correlation coefficients and level of significance for the traits of sesame. PH, Plant height; CH, first capsule height; CL, capsule length; ITL, infertile top length; SPC, seeds per capsule; CPP, capsules per plant; SW, 1000-seed weight; YPP, yield per plant; NCL, number of capsule layers; SFW, sesame fusarium wilt; SCC, seed coat color.

Phenotypic data were collected for 11 agronomic traits of sesame to examine the correlation among them. All traits exhibited normal distributions, with no significant skewness (**Figure 1A**). All the traits showed a small skewness and kurtosis values with low standard error. However, seeds per capsule stood out with high skewness and kurtosis values (**Supplementary Table S1**). Strong correlations were among between several traits (**Figure 1B**). For instance, there were significant positive correlations between 1000-seed weight (SW) and capsule length (CL), Number of capsule layers (NCL), and plant height (PH), NCL. There were positive correlations between capsules per plant (CPP), as well as yield per plant (YPP) and CPP. On the other hand, NCL and infertile top length (ITL) showed strong negative correlations (**Figure 1B**).

### Genome resequencing and genotyping

Parent ‘Yizhi’ produced a total of 10.35 Gb of clean data, and parent ‘Mingdeng Zhima’ produced 9.23 Gb of clean data. The total data volume of the 169 descendants was 486.10 Gb. The GC percentage ranged from 33.74% to 41.13% and the Q30 was above 88% (**Supplementary Table S2**).

The data reliability can be seen from a negligible single base error rate along the read position, which was maintained under 0.006% (**Supplementary Figure S1A**). The total GC content was well below 40% (**Supplementary Figure S1B**). Moreover, the AT and CG bases were basically not separated, and the curve was relatively flat, indicating that the sequencing results were normal (**Supplementary Figure S1B**).

The average depth of coverage of parents and offspring and the corresponding proportion of genome coverage are shown in **Supplementary Table S3**. It ccan be seen that the average coverage depth of the parent genome was more than 20X. The average coverage of the genome was more than 90% (at least 1X coverage). The average coverage depth of the offspring samples was 9.01X, and the coverage was more than 97.79% (at least 1X coverage). Poor coverage may be due to improper assembly of the reference genome or a distant relation to the parent, making it impossible to compare sequencing data to the reference genome. In general, the distribution of base coverage depth on the genome indicates a higher level of sequencing randomness.

### Construction of high-density linkage bin map and evaluation

**Figure 2:**
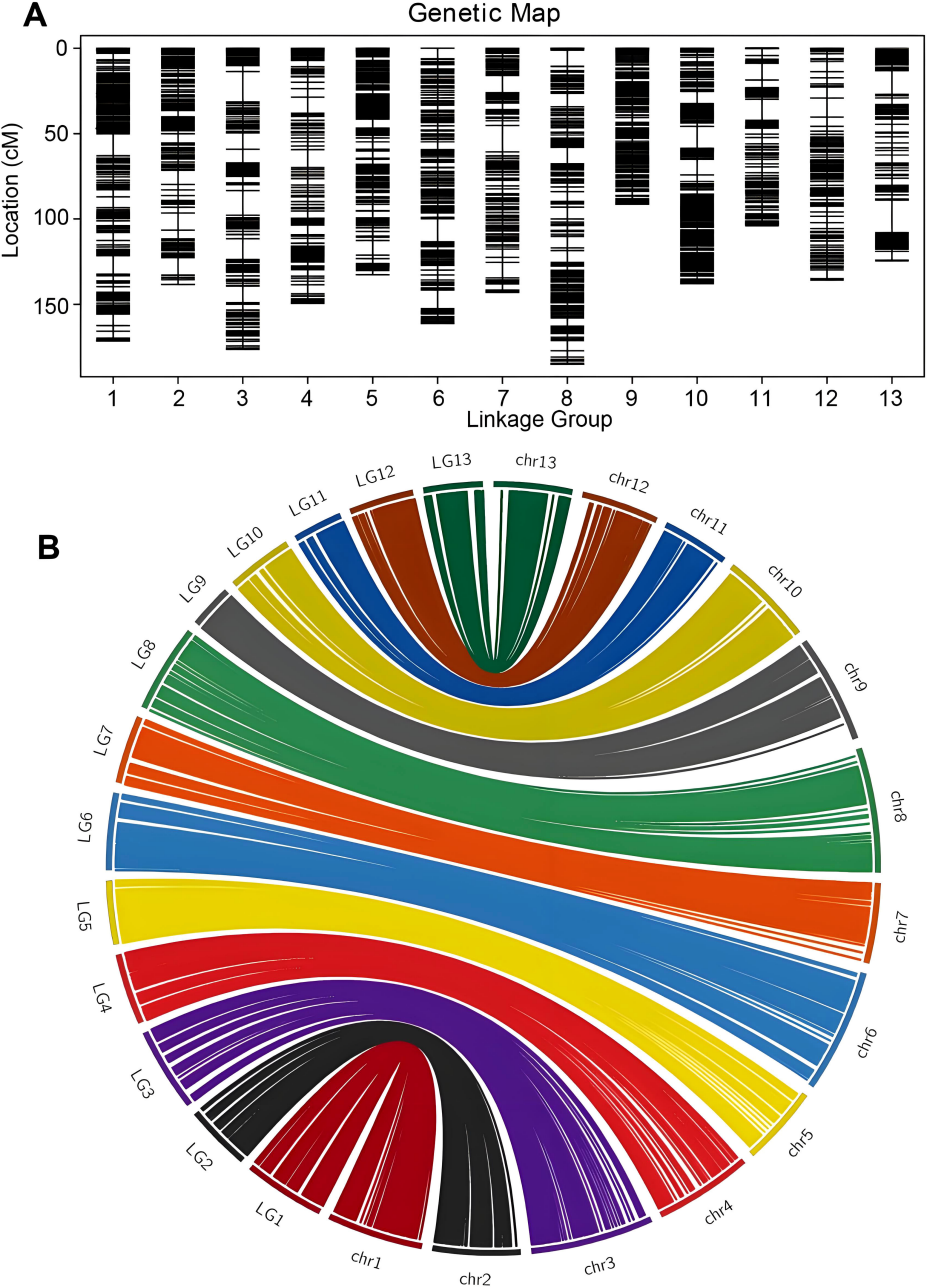
High-density genetic bin map construction based on genome resequencing and collinearity analysis of the markers between the genetic map and the physical map. **(A)** Distribution of markers with unique block loci in the genetic linkage bin map of sesame. **(B)** Syntenic analysis of makers between sesame genetic map and *Sesamum indicum* genome via circle diagram.

Based on the available information, a total of 2766 bins were divided into 13 linkage groups (**Figure 2A**). The HighMap software analysis enabled the determination of the linear arrangement of markers within each linkage group, and estimation of the genetic distance between adjacent markers. Ultimately, a genetic map with a total map distance of 1850.52 cM was obtained.

The statistics of the number of bins, total genetic distance, average genetic distance, maximum gap distance and proportion of gap less than 5 cM of each linkage group are shown in **Table 1**. Graphical genotype analysis was performed on 169 offsprings using 2766 bin loci, as shown in the **Supplementary Figure S2**. Most strains had a small number of chromosomes in their genomes that hadn’t undergone recombination, meaning they originated entirely from a single parental genome. There were also some strains with chromosomal segments that are heterozygous, which might be related to incomplete repair or incorrect repair after chromosome exchange.

**Table 1:** Summary of the bin map in sesame F2 population.

LG	nloc	nind	sum	gap_5	gap_5 (%)	max gap	Distance (cM)	aver. Distance (cM)
1	355	169	59995	350	0.9887	12.87	171.45	0.48
2	190	169	32110	184	0.9735	10.26	138.3	0.73
3	235	169	39715	228	0.9744	17.78	176.2	0.75
4	149	169	25181	143	0.9662	10.51	149.39	1
5	207	169	34983	204	0.9903	8.6	132.66	0.64
6	233	169	39377	229	0.9871	13.52	161.12	0.69
7	178	169	30082	174	0.9831	15.48	143.02	0.8
8	269	169	45461	260	0.9701	9.58	184.93	0.69
9	215	169	36335	214	1	2.81	91.26	0.42
10	282	169	47658	278	0.9893	12.88	137.93	0.49
11	156	169	26364	153	0.9871	12.19	103.97	0.67
12	155	169	26195	150	0.974	11.2	135.87	0.88
13	142	169	23998	138	0.9787	19.01	124.41	0.88

Collinearity analysis was performed by the position of the marker on the genome and the genetic map (**Figure 2B**). Spearman correlation coefficient of each linkage group and the physical graph showed the collinear quality (**Supplementary Table S4**). The closer the Spearman coefficient is to 1, the better the collinearity of the map and the physical map. The correlation coefficient values were all above 0.97, suggesting significant collinearity between linkage groups and physical map.

### Population SNP detection and annotation

A total of 1,09,696 alleles were identified among all the samples (**Supplementary Table S5**). The percentage of ‘aa’ genotype ranged from 44.82% to 84.24%. However, the ‘bb’ genotype was less common, accounting for 9.44%−52.25%. The ‘ab’ genotype showed a narrow range, varying from 2.39% to 23.26%. The rate of missing alleles ranged from 1.41% to 15.8%, indicating a relatively low proportion of missing alleles (**Supplementary Table S5**). The SNP variation had two types, including conversion and reversal. A total of 5,56,668 SNPs were detected with a conversion/reversal (Ti/Tv) ratio of 1.78 (**Supplementary Table S6**). The ratio of heterozygosity ranged from 21.48% to 68.16%.

By determining the location of the variant site on the reference genome and using gene position information, we identified the specific region in the genome where the variant occured. Additionally, this allowed us to determine the impact of mutation (**Supplementary Figure S3**). Most abundant SNP variants occurred in the intergenic (29.29%), upstream (27.83%) and downstream (22.50%) regions. The CDS contained 6.84% of SNP variant sites, which further included synonymous_coding (49.39%) and non-synonymous_coding (48.67%) regions as the effects of mutations (**Supplementary Figure S3A**). The major distribution sites for InDels included upstream (33.40%), downstream (24.51) and intergenic (24.13%) regions (**Supplementary Figure S3B**). However, compared to SNPs, only 2.17% of InDels were situated on CDS, which further covered frame_shift (67.68%), codon_deletion (9.18%), and codon_insertion (8.81%) as major effects of mutations (**Supplementary Figure S3B**).

#### Detection of QTLs associated with key agronomic traits

**Figure 3:**
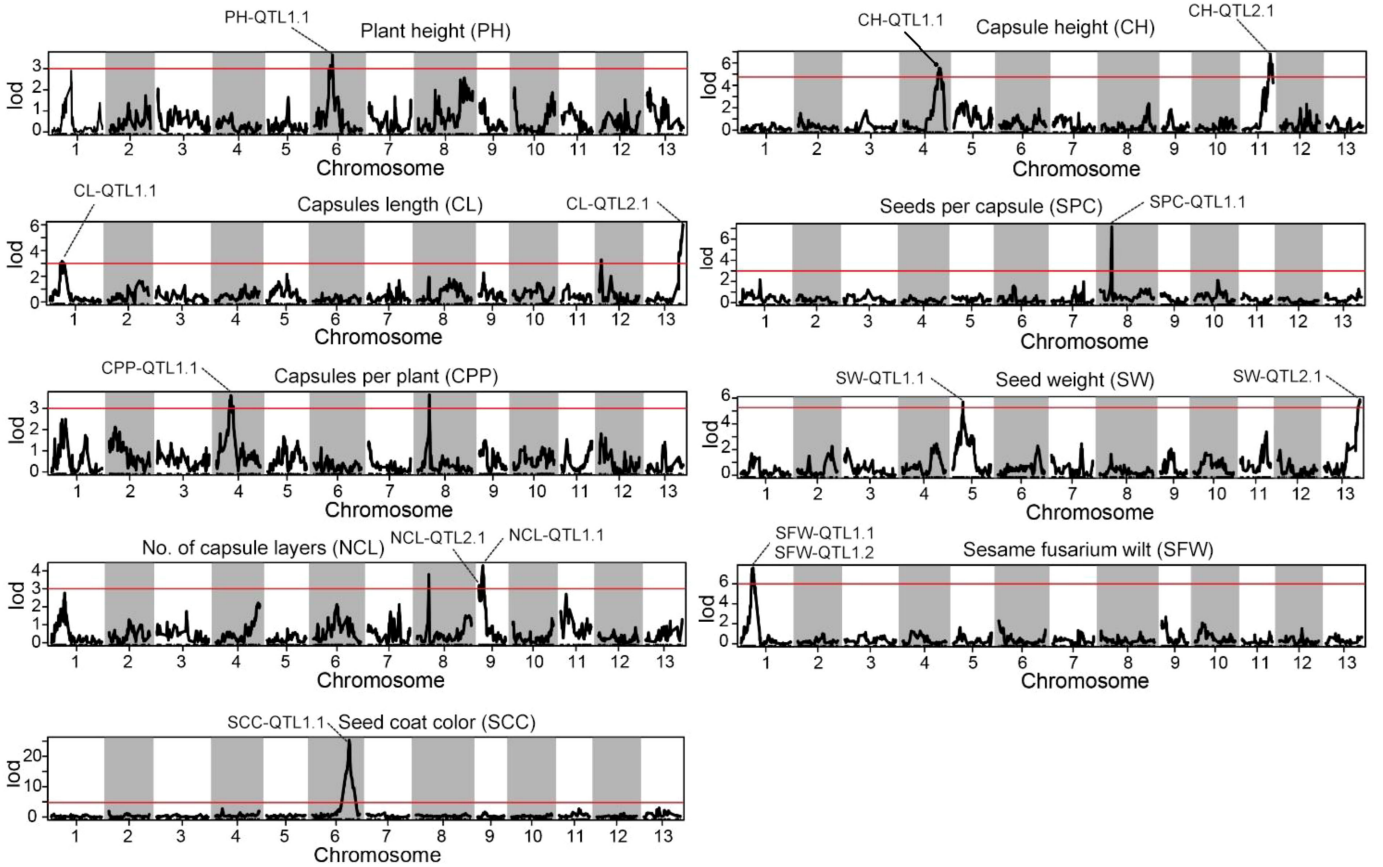
Distribution of mapping QTLs with ten different traits on the sesame genetic map. The red lines indicate confidence interval of QTLs with LOD score above threshold 3.0.

Statistical analysis method was used for the identification of significant QTLs that incorporates LOD (Logarithm of Odds) scores and confidence intervals. The text outlines a procedure for using the IM interval mapping method to locate traits. The LOD thresholds were set based on the results of permutation test performed 1000 times. The process considers different confidence levels (0.99, 0.95, and 0.90) for LOD thresholds. If a mapping interval could not be found, the text recommends manually adjusting the threshold. It suggested starting from 3.0 and gradually decreasing it to 2.5 or 2.0 if necessary. Using a threshold of 3, we identified 14 significant QTL sites associated with the agronomic traits of *S. indicum* (**Table 2**; **Figure 3**). SS, SG, SW, NCL, and SFW each contained two potential QTL sites; while each of the remaining trait possessed one site. The PVE (phenotypic variance explained) for these QTL sites ranged from 9.16% for NCL-QTL1.1 to 18.23% for SPC-QTL1.1 (**Table 2**). Except for NCL-QTL1.1, all other QTL sites showed a PVE greater than 10%. SFW-QTL1.1 and SFW-QTL1.2 showed a significant PVE above 20%, while SCC-QTL1.1 had a PVE of 52.72%. The QTL distribution over the genome was not uniform. Among the 14 potential QTL sites, three were located on chromosome 1, whereas each of the chromosome 4, 6, 9 and 13, contained 2 potential QTL sites.

**Table 2:** List of QTLs for eight traits identified in sesame F_2_ population.

Trait	QTL name	LOD Threshold	Group ID	Start (cM)	End	Peak position (cM)	MaxLOD	ADD	DOM	PVE(%)
PH	PH-QTL1.1	3.2	6	61.761	62.648	62.057	3.649	6.225	-1.108	15.233
CH	CH-QTL1.1	4.5	4	118.264	129.633	124.538	5.554	-2.938	-0.863	13.852
CH	CH-QTL2.1	4.7	11	89.937	102.782	94.713	6.765	-3.362	-0.218	17.906
CL	CL-QTL1.1	3.0	1	34.301	45.547	39.985	3.153	-0.093	0.176	11.017
CL	CL-QTL2.1	4.1	13	110.185	124.408	124.112	6.010	-0.169	0.045	18.621
SPC	SPC-QTL1.1	3.0	8	37.529	39.425	39.425	7.166	32.874	-31.521	18.259
CPP	CPP-QTL1.1	3.0	4	45.176	50.728	49.456	3.605	-6.933	8.873	10.384
SW	SW-QTL1.1	4.7	5	33.403	37.909	36.069	5.704	-0.110	-0.027	14.658
SW	SW-QTL2.1	4.9	13	116.698	124.408	124.112	5.881	-0.121	-0.001	17.588
NCL	NCL-QTL1.1	3.0	9	0.357	1.009	1.009	3.192	-1.857	0.691	9.159
NCL	NCL-QTL1.2	3.0	9	9.828	17.045	14.078	4.287	-2.109	1.832	13.828
SFW	SFW-QTL1.1	6.5	1	36.372	39.985	37.912	7.503	11.909	-15.298	24.852
SFW	SFW-QTL1.2	6.5	1	41.169	44.068	41.760	7.588	11.276	-16.051	23.839
SCC	SCC-QTL1.1	22.2	6	123.487	127.109	125.270	25.294	-1.529	2.059	52.720

### Identification of overlapping QTLs associated with SFW and SCC traits

**Figure 4:**
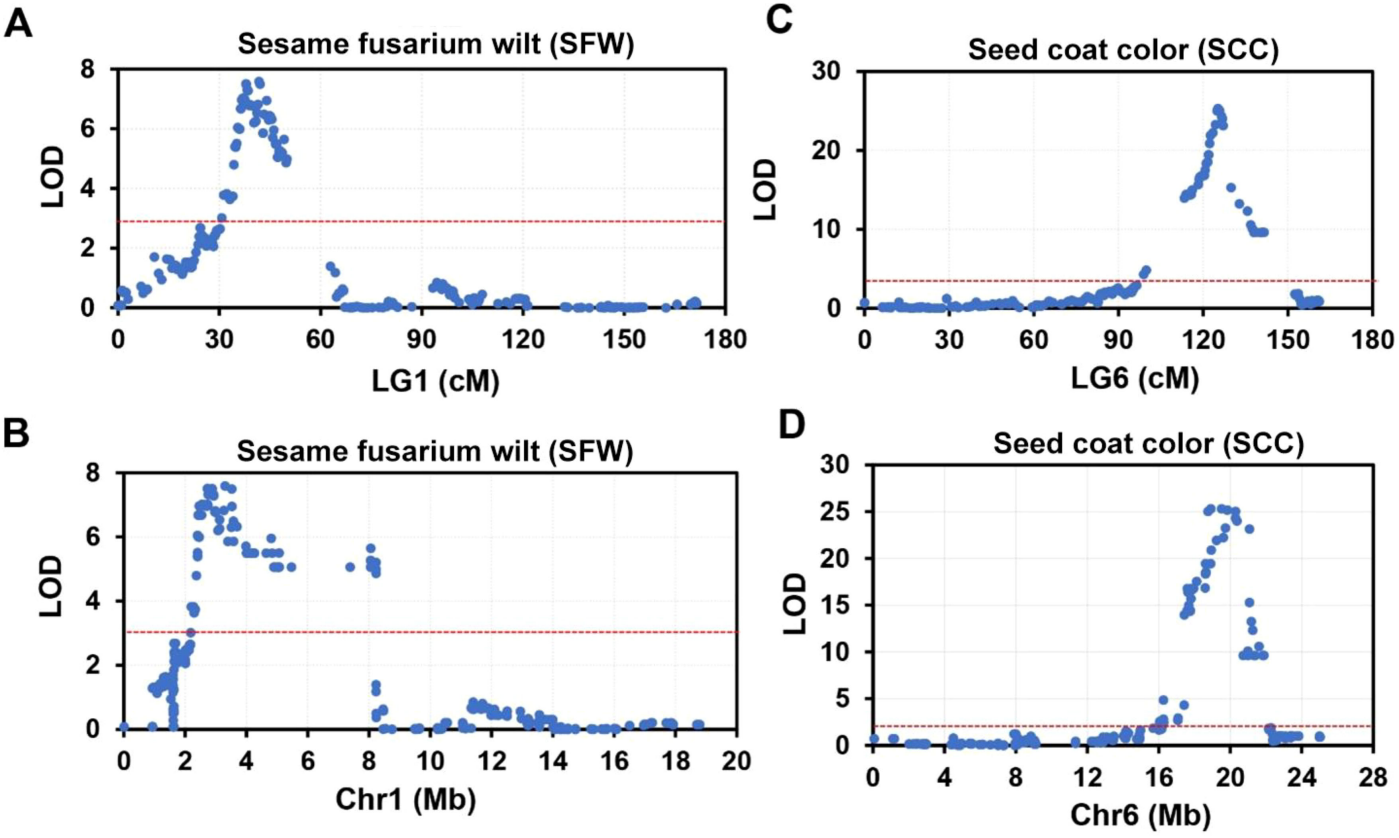
The top two significant QTLs for sesame fusarium wilt (SFW) and seed coat color (SCC) on linkage group (LG) 1 and LG6. **(A, B)** Significant QTLs responsible for SFW trait on LG 1 **(A)** and its corresponding physical position on chromosome (Chr) 1 **(B)**. **(C, D)** Significant QTLs responsible for SCC trait on LG 6 **(C)** and its corresponding physical position on Chr6 **(D)**.

Two most significant QTLs were identified for sesame fusarium wilt and seed coat color. Two major QTLs of sesame fusarium wilt (SFW-QTL1.1 and SFW-QTL1.2) were located on chromosome 1, each explaining 23.84%−24.8% of the PVE, with an LOD score above 6. These QTLs contained 28 bin markers from locus 121 to 153 (**Supplementary Table S7**). QTL1.1 occupied 20 loci and the QTL1.2 engaged the remaining 8 loci. The other most important trait was seed coat color with one QTL (SCC-QTL1.1), which was also located on chromosome 1. SCC-QTL1.1 had the highest PV above 50%. This SCC-QTL1.1 contained 11 intervals (Block2445-Block2403) (**Supplementary Table S7**). However, decreasing the threshold to 3 increased the number of QTLs for sesame fusarium wilt and seed coat color, each accounting for more than 30 QTL bin makers (**Figure 4**).

### Identification and annotation of genes in the QTL intervals

**Figure 5:**
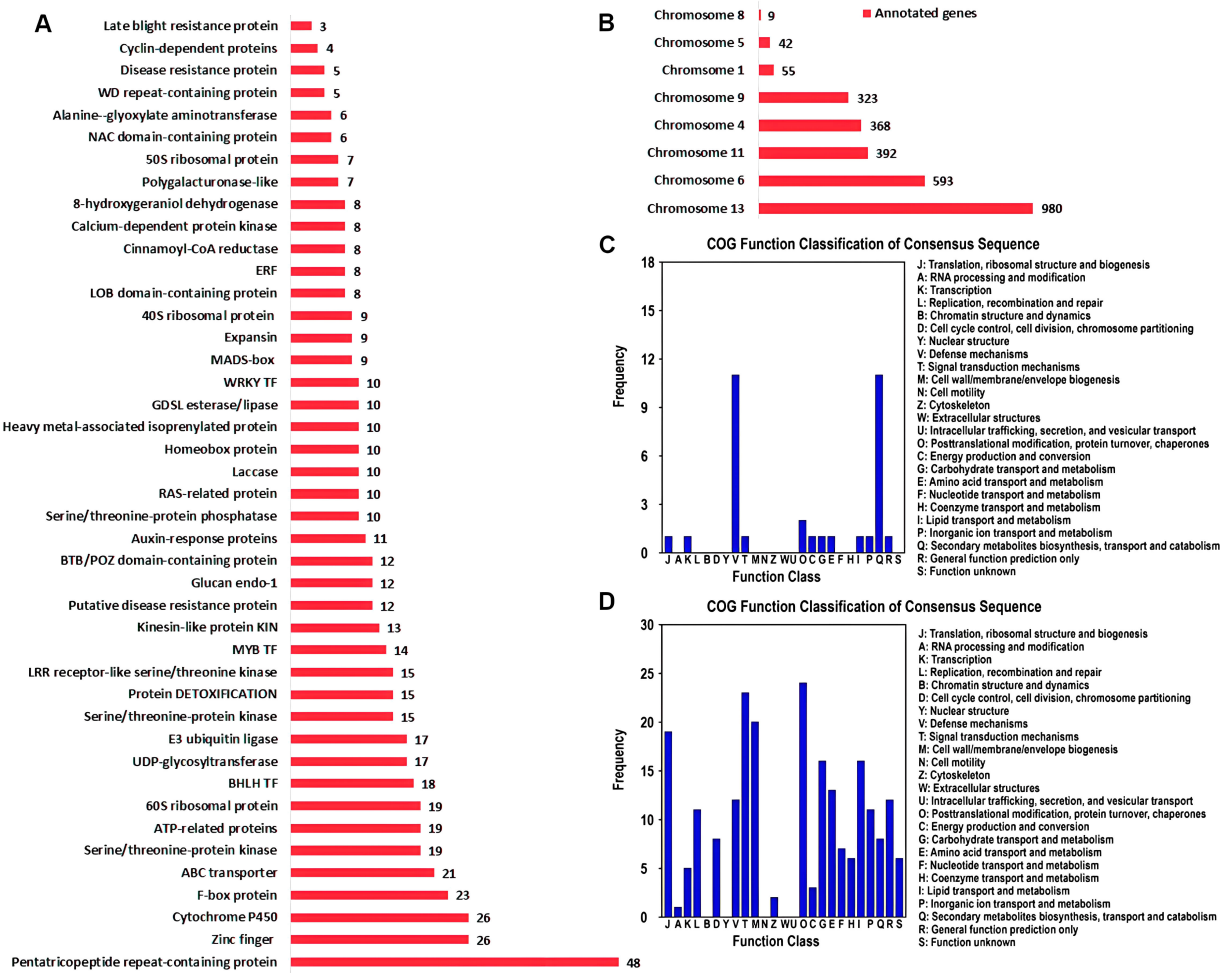
Functional enrichment of genes located in the QTL intervals. **(A)** NR annotation summary showing the most abundant gene families. **(B)** The frequency of annotated genes on individual chromosome. **(C)** COG function classification of genes in SFW-QTL1.1 and SFW-QTL1.2 QTL intervals related to SFW trait. **(D)** COG function classification of genes in SCC-QTL1.1 related to SCC trait.

The gene sets associated with QTL intervals were annotated using different annotation platforms and NR annotations of 2762 genes were obtained (**Supplementary Table S8**). Pentatricopeptide repeat-containing (PPR) proteins were the most abundant annotations among the annotated genes (**Figure 5A**). PPR proteins regulate plant defense-pathway genes by modulating RNA processing and translation and fine tune the expression of stress-responsive genes. Cytochrome P450 (CYPs) and zinc finger proteins were the second most abundant categories. Regarding the disease resistance, CYPs played important roles. These enzymes involved the biosynthesis of phytoalexins, which were potential secondary metabolites helping plants to cope with pathogenic attack. Moreover, CYPs also contribute to the detoxification of dangerous compounds produced by pathogens and regulate plant defense responses. Among the other categories, protein DETOXIFICATION, LRR receptor-like serine/threonine kinase, putative disease resistance protein, disease resistance protein and late blight resistance protein were the most important gene annotations for plant disease control (**Supplementary Table S8**; **Figure 5A**). The annotated genes were located on 8 chromosomes (**Figure 5B**). Of the 2762 genes, 980 were associated with the QTL intervals positioned on chromosome 13 and 593 were linked with chromosome 6. The lowest were allied with chromosome 8, containing only 9 genes.

sesame fusarium wilt and seed coat color were identified as the most important QTLs and functional annotation of the genes associated with these QTLs were significantly involved in plant immune responses and signal transductions (**Figures 5C, D**). Two distinct annotation categories were observed in the genes connected with SFW-QTL1.1 and SFW-QTL1.2 (**Figure 5C**). These were the ‘defense mechanisms’ and ‘secondary metabolite biosynthesis, transport and catabolism’, suggesting that SFW could specifically be involved in plant defense and immunity responses. However, multiple regulatory processes were linked with SCC-QTL1.1, including ‘signal transduction mechanism’, ‘posttranslational modification, protein turnover, chaperones’, ‘cell wall/membrane/envelope biogenesis’, ‘translation, ribosomal structure and biogenesis’, ‘lipid transport and metabolism’ and ‘defense mechanisms’ (**Figure 5D**). This suggested a multi-directional role of SCC-QTL1.1.

## Discussion

### Genetic analysis of Fusarium wilt defense- and yield-related traits

Plant height, capsule characteristic, yield per plant, and sesame Fusarium wilt disease are important agronomic traits. We used F_2_ population from parents with significant differences in traits for an intraspecific cross, and a new sesame high-density genetic map was constructed with 2766 bin loci and identified significant QTLs. For precise QTL mapping results, we performed segregation analysis with a large experimental group and multiple phenotypic indexes. The frequency distribution of phenotypic characters was analyzed, revealing a normal distribution and a significant correlation between different traits. This suggests a polygene mode of genetic control.

Significant positive correlations (P ≤ 0.05) were observed among NCL, PH, CL, CPP, SW. Meanwhile, sesame fusarium wilt related trait SFW was negatively correlated with PH, CL, SW, NCL (P ≤ 0.05), while it was positively correlated with infertile top length (ITL). Plant height, capsule length, capsules per plant, 1000-seed weight are important factors of sesame yield composition (Monpara, 2016). We analyzed the correlation between the eleven important traits. The results showed that plant height, seeds per capsule, and capsules per plant were positively correlated with yield per plant (P ≤ 0.05).

The high-yield and disease-resistant varieties are important achievements in sesame breeding, increasing food security. Fusarium wilt of sesame, caused by *Fusarium oxysporum* f. sp. sesami, is a destructive soil-born fungal disease that causes huge economic losses in China (Zhang et al., 2001). Developing a high-yielding, stress-resistant variety is a viable option to address these challenges. Previous studies about Fusarium wilt mainly focused on the molecular characterization of *F*. *oxysporum* f. sp. Sesami, field trials evaluating and screening sesame resistance to fusarium wilt (Herrera and Laurentin, 2014; Silme and ÇAĞIrgan, 2010; Jyothi et al., 2011; Li et al., 2012). In the genetic and genomic analysis, seven positive markers (five RAPD and two ISSR) linked to Fusarium wilt resistance were found in the line C3.8 (Anter and Ghada, 2021). Transcriptome comparison of resistant and susceptible sesame varieties inoculated with *F*. *oxysporum* f.sp.sesami showed that phenylpropanoid biosynthesis pathway may play more important role in Fusarium wilt resistance in early stage infection (Wei et al., 2016). A genome-wide association study (GWAS) of an interspecific population and genome comparisons revealed a long terminal repeat insertion and a sequence deletion in DIR genes of wild *Sesamum angustifolium*, while the cultivated sesame independently cause high susceptibility to Fusarium wilt disease (Miao et al., 2024). A study on the inheritance of resistance to Fusarium wilt in sesame indicated that the genetic effect of Fusarium wilt resistance was complex and controlled by dominant, additive, and recessive effect (Wang, 1993; El-Bramawy, 2006).

In the F_2_ population, we investigated the segregation of individuals in Fusarium wilt disease resistance. Based on the phenotyping and linkage map, QTLs were identified in LG1, suggesting their important role in disease resistance. The number of genes regulating the Fusarium wilt disease varies in different species of sesame, and different genes and genetic variations are detected for controlling the trait (Wei et al., 2016; Miao et al., 2024). The genetic mechanism of Fusarium wilt disease is still unclear.

### Genetic linkage map

QTL mapping is an important method applied to locate the candidate genes controlling specific traits. Genotyping-by-sequencing (GBS) is a high-throughput technique employed to develop SNP markers in a short time for high-density genetic map construction and QTL mapping. To date, this technique has been widely used for locating the key traits in sesame (Zhang et al., 2013; Wu et al., 2014; Du et al., 2019; Yol et al., 2021).

In this study, we constructed a high-density bin map for sesame using the whole genome resequencing approach, achieving mass identification of SNPs and InDels for sesame. We discovered 5,56,668 SNPs. Most of SNPs occurred in the noncoding region of the genome, including intergenic (29.29%), upstream (27.83%) and downstream (22.50%) regions. While only 6.84% of SNPs were located in protein coding region, including synonymous_coding (49.39%) and non-synonymous_coding (48.67%) as the effects of mutations. This new map has several advantageous features compared to other published genetic maps in sesame. Firstly, larger F_2_ segregating populations of 169 individuals were used. Secondly, the map is an ultra-high-density genetic map with 2766 bins, including 1,10,345 SNPs with a sequencing depth more than 4×. Thirdly, the 13 LGs (linkage groups) corresponded to the 13 chromosomes in the sesame karyotype (2n = 26), allowing for precise gene mapping. Lastly, the estimated size of the sesame genome is 1850.52 cM with an average marker distance of 0.67 cM per bin. The marker density is significantly higher than that of previously published maps. Therefore, the map in this study is superior to previously published sesame maps and it can contribute to the development for QTL analysis, gene mapping, and map-based cloning.

### QTL identification of seed-related traits

In this study, QTL analysis of 11 important traits in sesame (ten yield related traits and one Fusarium wilt disease evaluation) identified 14 associated regions. All of these QTLs contributed above 10% of the phenotypic variation with LOD > 3, except for NCL-QTL1.1. Eleven QTLs were related to yield, two QTLs were associated with disease resistance, and one QTL was associated with seed coat color.

In the QTLs for yield, PH-QTL1.1, CH-QTL2.1, CL-QTL2.1, SPC-QTL1.1, SW-QTL2.1 played major roles, explaining more than 15.0% of the phenotypic variation. SFW-QTL1.1 and SFW-QTL1.2 are responsible for Fusarium wilt, and explained 24.9% and 23.8% phenotypic variation. These Fusarium wilt related QTLs are reported for the first time in sesame, and their genetic control was mostly comprised of major QTLs with R2 ≥ 20%. An important QTL of seed coat color was detected in LG6 with LOD value equal to 22.2, explaining 52.7% of phenotypic variation. Similar to previous study (Zhang et al., 2013; Wei et al., 2015; Wang et al., 2016; Du et al., 2019; Li et al., 2021), our results also showed that seed color of sesame was controlled by major QTL. However, the number of QTLs regulating seed colors is different among these studies. Zhang et al. (2013) detected four QTLs on three LGs controlling the seed coat color using an F_2_ population (Zhang et al., 2013). Wei et al. (2015) identified seven QTLs distributed on three LGs using GWAS strategy (Wei et al., 2015). Du et al. (2019) found seven seed coat color related QTLs on three LGs (Du et al., 2019). However, only one major QTL on LG6 was identified, suggesting that seed coat color is controlled by multiple major QTLs and their genetic mechanism might be different.

### Candidate gene function analysis

In total, there were 2762 candidate genes in the confidence interval with R2 ≥ 9% (**Supplementary Tables S8**). For yield-related trait, there were 22 functional categories in COG analysis. Carbohydrate transport and metabolism, signal transduction mechanisms, and lipid transport and metabolism harbored a large number of candidate genes. In KEGG analysis, the largest number of genes were enriched in biosynthesis of secondary metabolites and carbon metabolism, harboring 69 genes and 19 genes, respectively. SFW-QTL1.1/SFW-QTL1.2 and SCC-QTL1.1 were identified as the most significant QTLs involved in plant immune responses and signal transduction (**Figures 5C, D**). KEGG analysis showed two key genes involved in plant-pathogen interaction. One was annotated as putative late blight resistance protein R1A-4 (gene.SIN_1021452), and another was annotated as Rust resistance kinase Lr10 (gene.SIN_1021460). We suggest that genes in SFW-QTL1.1 loci may also play an important role in Sesame Fusarium wilt resistance.

In addition, pentatricopeptide repeat-containing (PPR) proteins were the most abundant annotations among the annotated genes (**Figure 5A**). PPR proteins regulate plant defense-pathway genes by modulating RNA processing and translation and fine tune the expression of stress-responsive genes. Recently, studies indicated that PPR proteins are essential for plant disease resistance, such as *Fusarium pseudograminearum* in wheat (Wang et al., 2021), *Ralstonia solanacearum* in potato (Park et al., 2016). Moreover, several other genes, including CYPs, DETOXIFICATION, LRR receptor-like serine/threonine kinase, also contribute to the detoxification of dangerous compounds produced by pathogens and might be involved in regulating plant defense responses. The signaling pathways of plant hormones also exhibit cross-talk in plant defense responses, which is an important and efficient strategy to resist the invasion of pathogens (Robert-Seilaniantz et al., 2007). These genes could contribute to disease resistance, plant growth, and ultimately yield-related traits. Our study has identified the QTLs and candidate genes. However, further study is needed to verify the markers nearest to the genes and their functions.

## Conclusions

This study is the first to map QTLs for yield-related traits, seed color, and Sesame Fusarium wilt disease in sesame using an F2 population. A high-density map was constructed using 3129 SNP markers. QTL analysis revealed 13 major-effect QTLs, with ten related to yield, two related to Sesame Fusarium wilt disease, and one related to seed color. These QTLs individually explained more than 10% of the phenotypic variation with LOD > 3. Additionally, three QTLs were identified with similar regions and partially explained the correlations among disease-related traits. Two key genes controlling Sesame Fusarium wilt disease were found in the SFW-QTL1.1 and SFW-QTL1.2 intervals (R2 ≥ 20%). This study establishes a strong foundation for further genetic analyses of Fusarium wilt disease related traits in sesame, including map-based gene cloning and marker-assisted selection breeding.

## Data Availability

The data presented in the study are deposited in the National Genomics Data Center (https://ngdc.cncb.ac.cn/) repository, accession number PRJCA025796 and in the Figshare (https://figshare.com/) repository, accession number figshare.27038404 (https://doi.org/10.6084/m9.figshare.27038404).
