# Supplementary material for: High-density genetic map construction and QTL mapping to identify genes for blight defense- and yield-related traits in sesame (Sesamum indicum L.)
Source: Front Plant Sci. 2024 Sep 26;15:1446062. doi: 10.3389/fpls.2024.1446062 (PMC11464332; doi:10.3389/fpls.2024.1446062)
Supplement: Supplementary file 1 [file DataSheet1.docx]

Supplementary Material

# 1 Supplementary Figures and Tables

## 1.1 Supplementary Figures


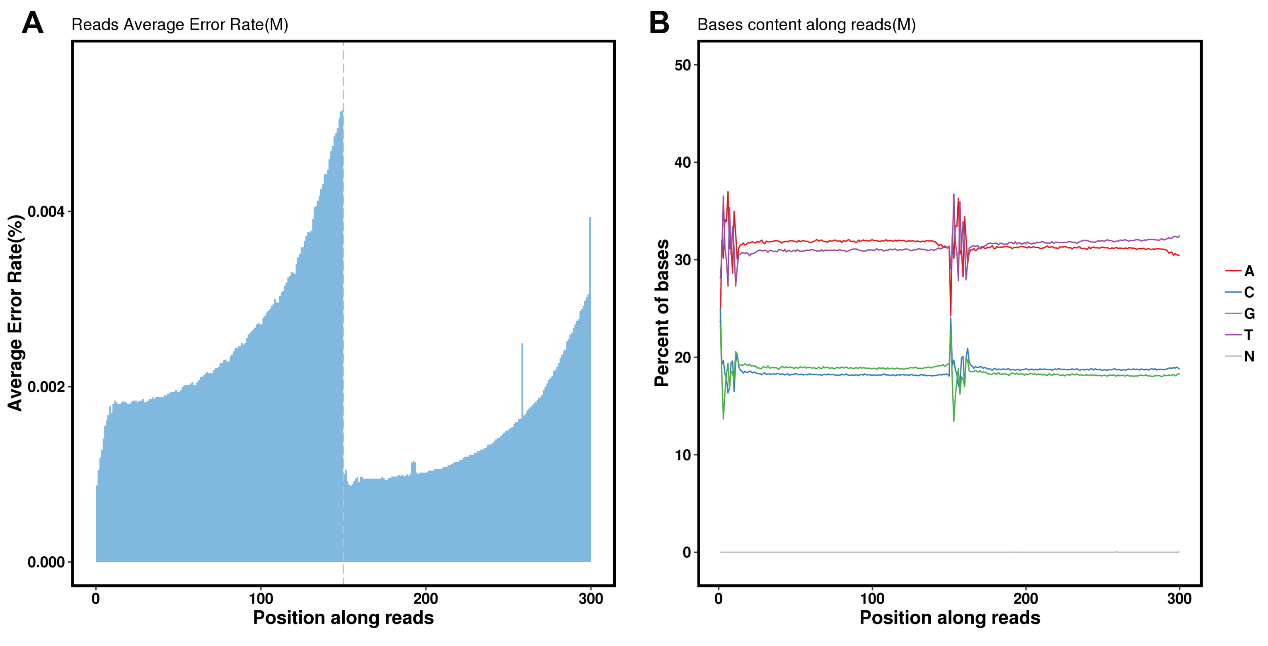


Figure S1 Test of the quality of sequencing data. (A) Average error rate of reads. (B) Percentage of A, T, C, G bases.


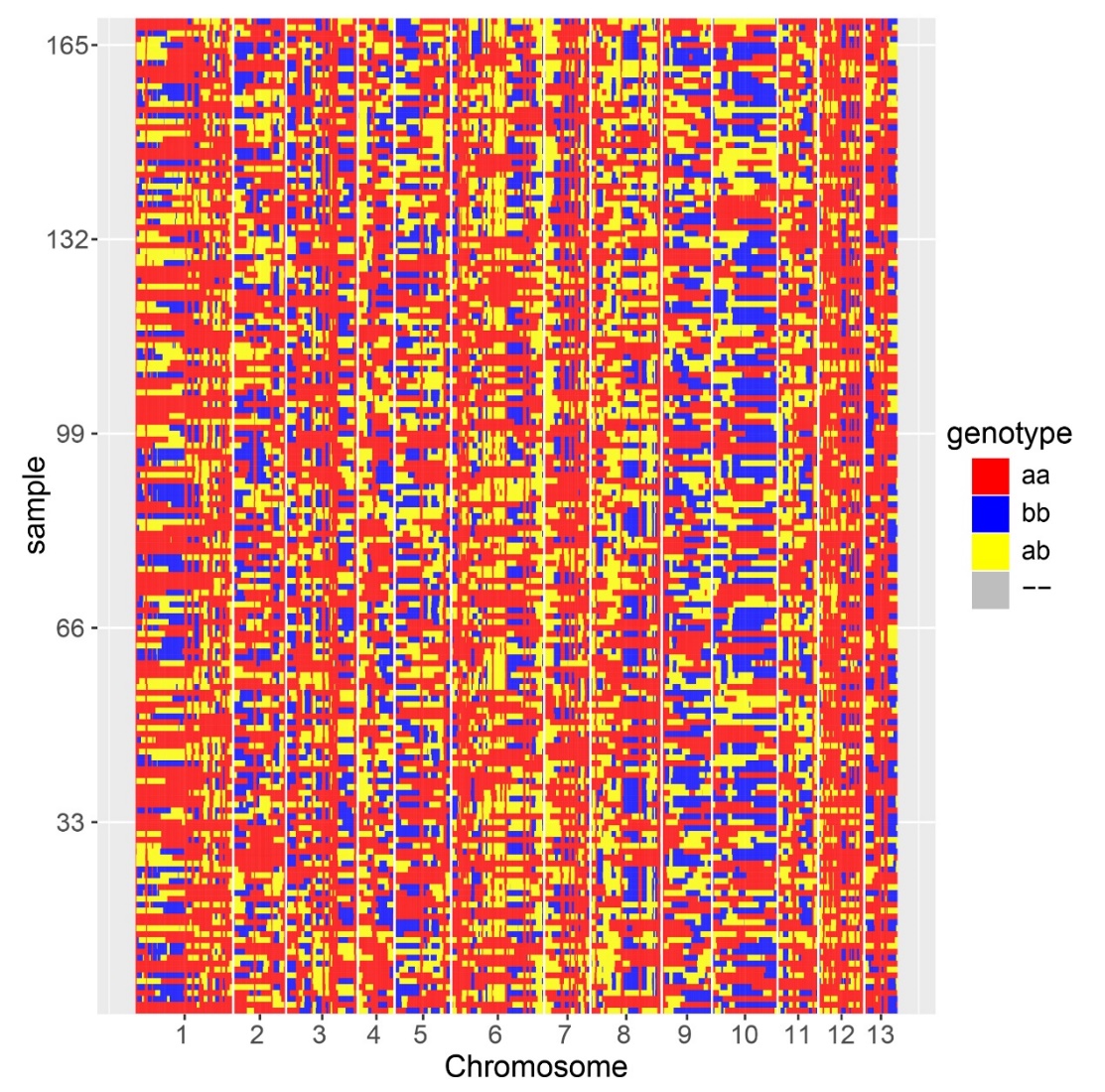


Figure S2. Illustration of the 2766 bin genotype from the 169 F2 individuals.


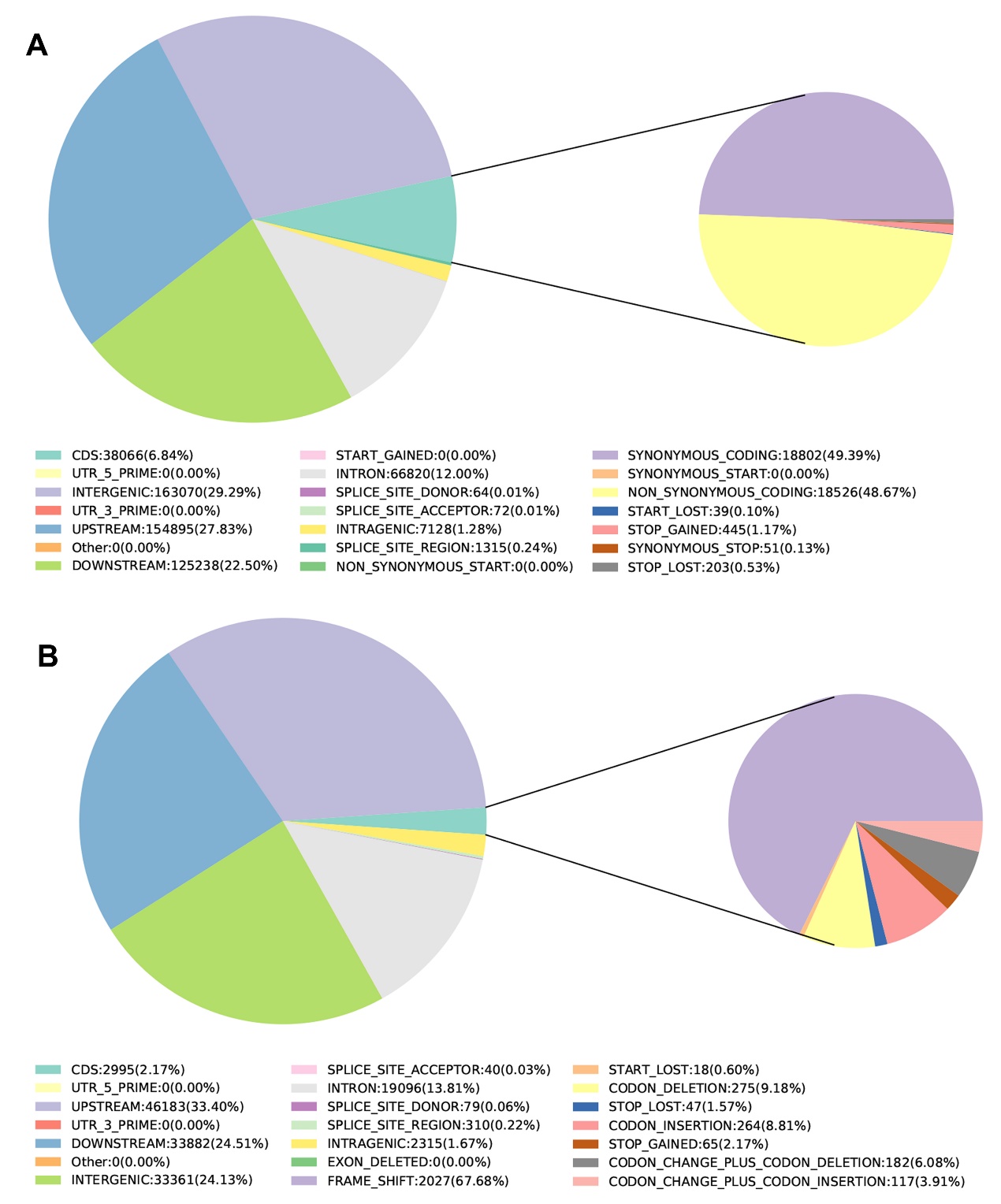


Figure S3.Annotation of genomic variants in the sesame F2 population. (A) Annotation of SNPs. (B) Annotation of Indels.


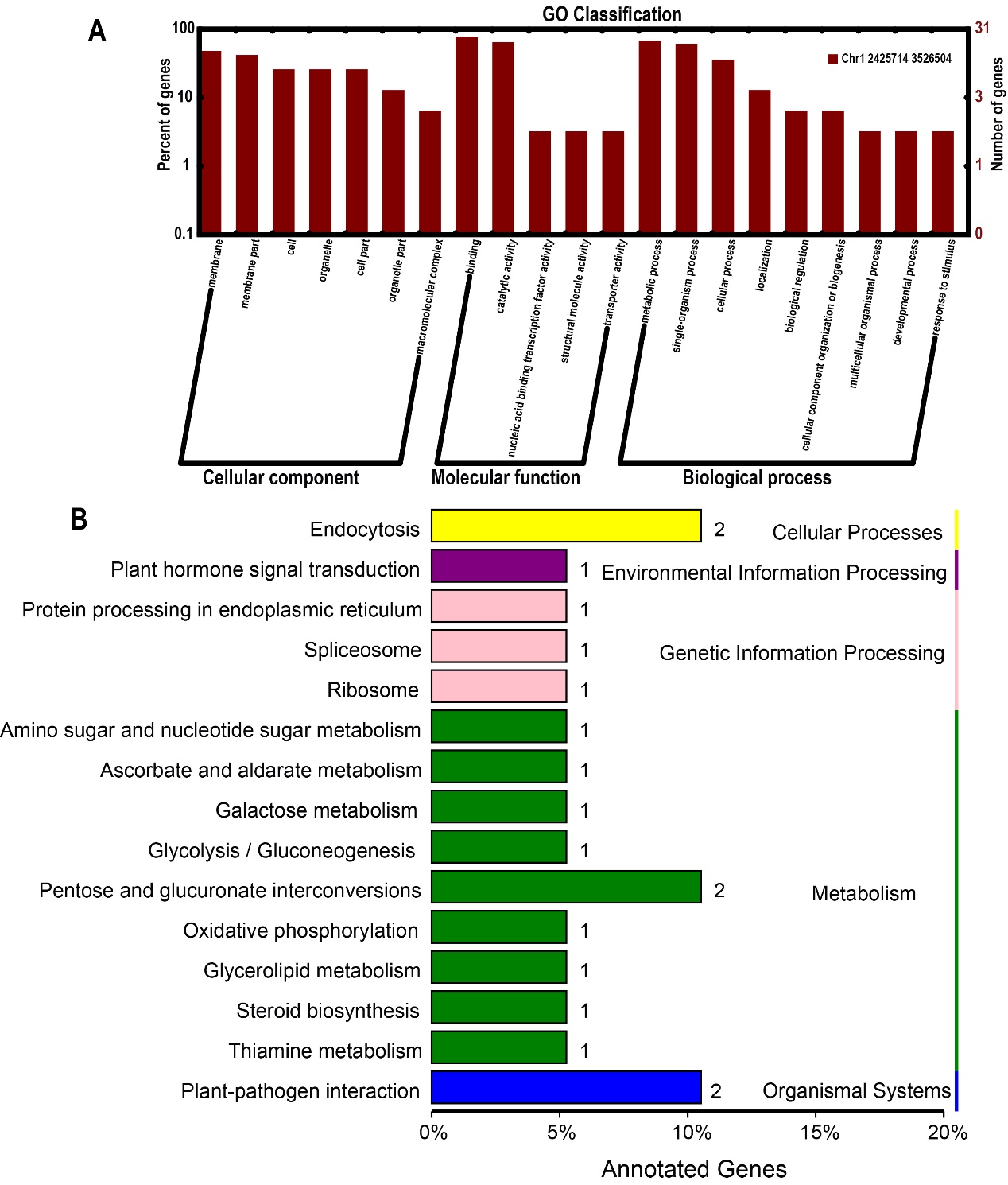


Figure S4. Functional enrichment of genes located SFW-QTL1.1 interval related to S based on Gene Ontology (GO) classification (A) and KEGG pathway enrichment (B).


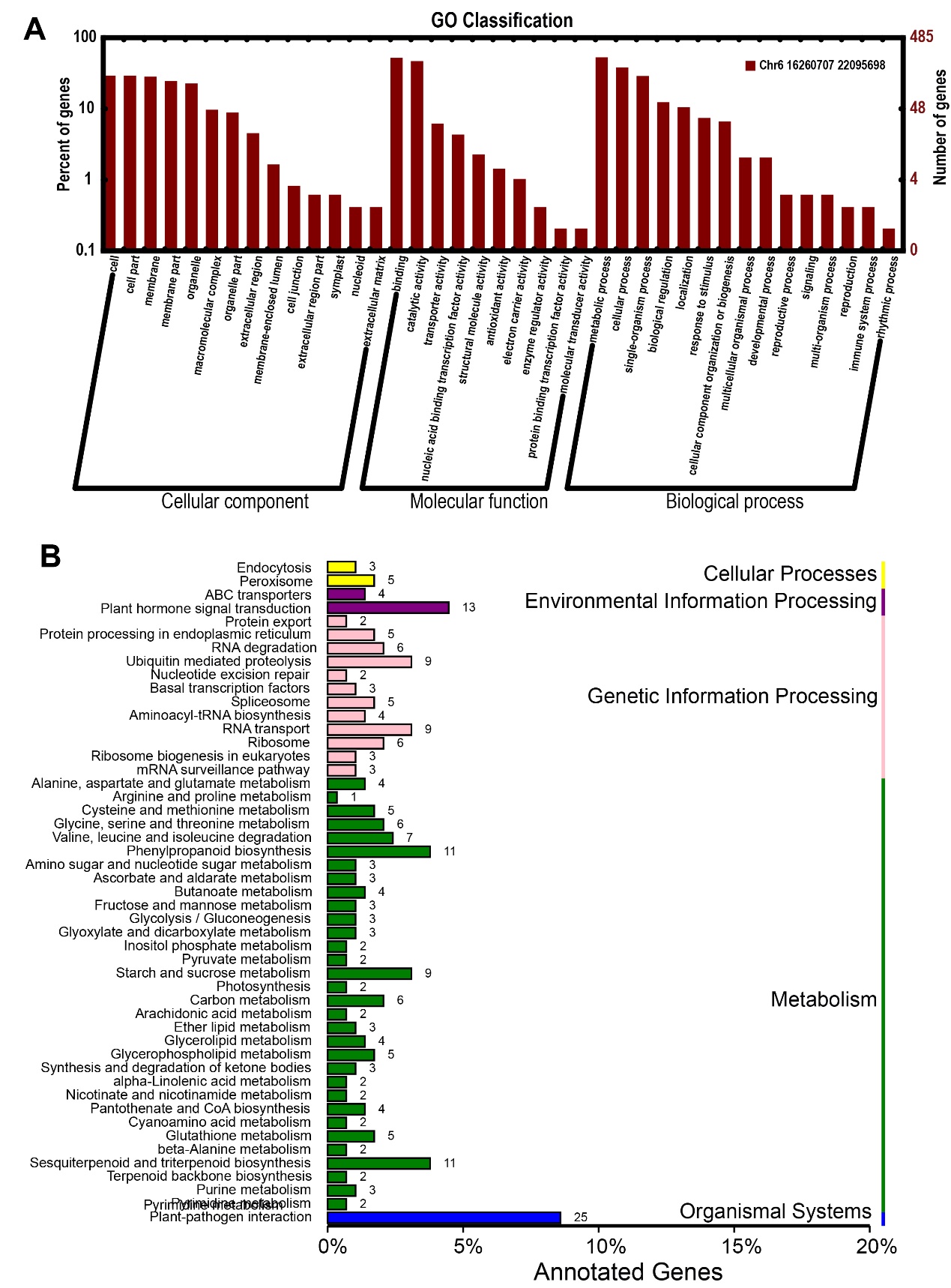


Figure S5. Functional enrichment of genes located ZP-QTL1.1 interval responsible for SCC trait based on Gene Ontology (GO) classification (A) and KEGG pathway enrichment (B).

## 1.2 Supplementary Tables

Table S1. Analysis of phenotypic vairation of eleven traits for the sesame F2 population.

Table S2. Accessment of sequencing data quality for each individuals.

Table S3. Detailed information of the sequencing data.

Table S4. Correlation coefficients for each linkage group.

Table S5. Statistics of genotypic ratio for each individual.

Table S6. Statistics of SNPs for the parents and each individual.

Table S7. Identification of significant markers in the QTL intervals with LOD score more than 3.0.

Table S8. Functional annotation of genes located in significant QTL intervals for eleven traits.

Table S9. Significant QTLs loacted in LG1 and LG6 responsible for SFW trait and SCC trait, respectively. .

Table S10. Functional annotation of genes located in SFW-QTL1.1 and SCC-QTL1.1.

Table S11. The growth stages and developmental characteristics of sesame.

Table S12. The methods or defined criteria for measuring 11 sesame phenotypic traits.
